# Supplementary material for: Histone H3 clipping is a novel signature of human neutrophil extracellular traps
Source: eLife. 2022 Oct 25;11:e68283. doi: 10.7554/eLife.68283 (PMC9665850; doi:10.7554/eLife.68283)
Supplement: Figure 2—source data 1. [file elife-68283-fig2-data1.zip › Figure 2-Source data 1-H4C.pdf]

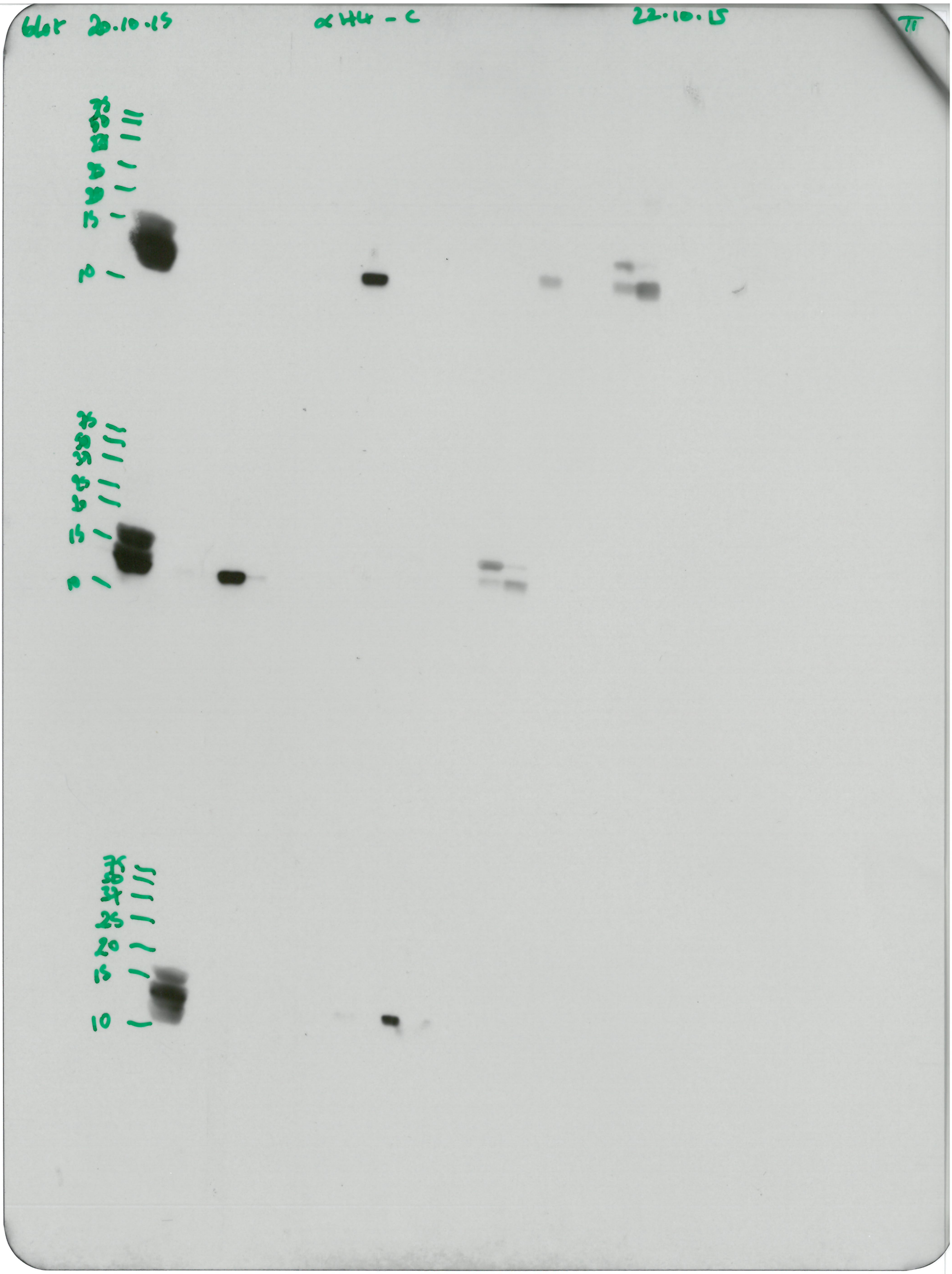

blot 20.10.15
αH4-C
22.10.15
15
10
25
20
15
10
