# Supplementary figures and images for: Histone H3 clipping is a novel signature of human neutrophil extracellular traps
Source: eLife. 2022 Oct 25;11:e68283. doi: 10.7554/eLife.68283 (PMC9665850; doi:10.7554/eLife.68283)

blot 20.10.15

21.10.15 TT

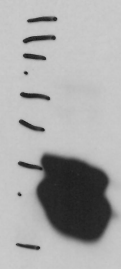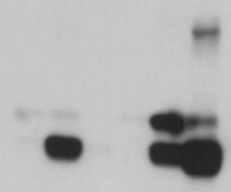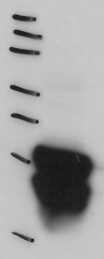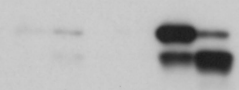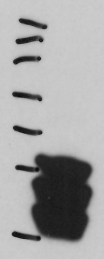

Supplement: Figure 2—source data 1. [file elife-68283-fig2-data1.zip › Figure 2-Source data 1-H3C.pdf]

blot 20.10.15

21.10.15 TT

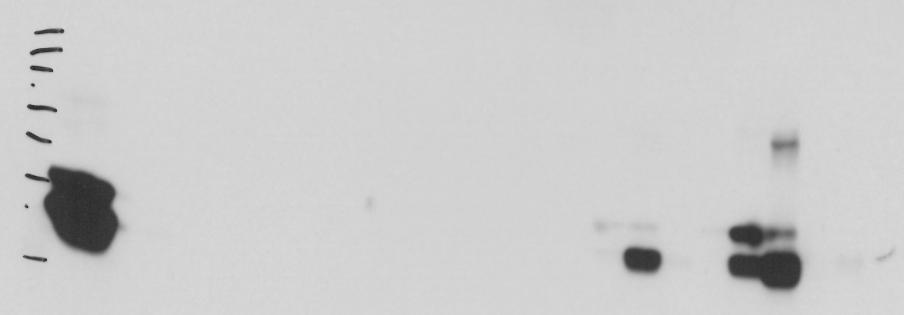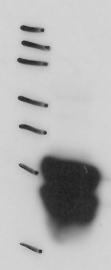

PMA 90 min  
stimulation

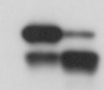

H3 fractions (H3C)

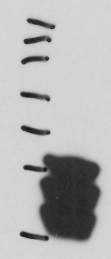

Supplement: Figure 2—source data 1. [file elife-68283-fig2-data1.zip › Figure 2-Source data 1-H3C_annotated.pdf]

6hr 20.10.15

$\alpha$  H4 - C

22.10.15

71

30  
25  
20  
15  
10  
5

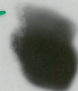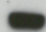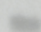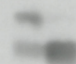

30  
25  
20  
15  
10  
5

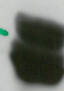

H4-C

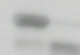

Remaining signal from H3-C probe

PMA 90 min  
stimulation

30  
25  
20  
15  
10

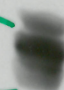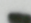

Supplement: Figure 2—source data 1. [file elife-68283-fig2-data1.zip › Figure 2-Source data 1-H4C_annotated.pdf]

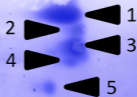

Supplement: Figure 2—source data 3. [file elife-68283-fig2-data3.zip › Figure 2-Source Data 5-Related to Figure 2B_annotated.pdf]

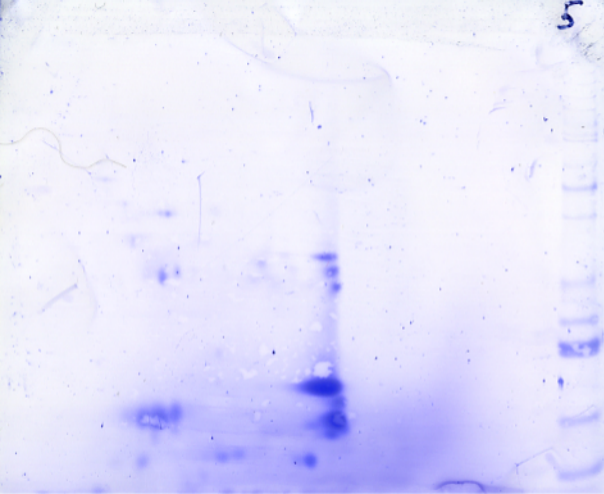

Supplement: Figure 2—source data 3. [file elife-68283-fig2-data3.zip › Figure 2-Source Data 5-Related to Figure 2B_original.pdf]

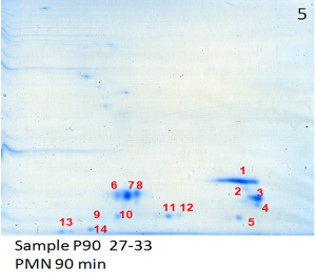

Supplement: Figure 2—figure supplement 2—source data 1. [file elife-68283-fig2-figsupp2-data1.zip › Figure 2-Figure supplement 2-Source data 1 .tif]

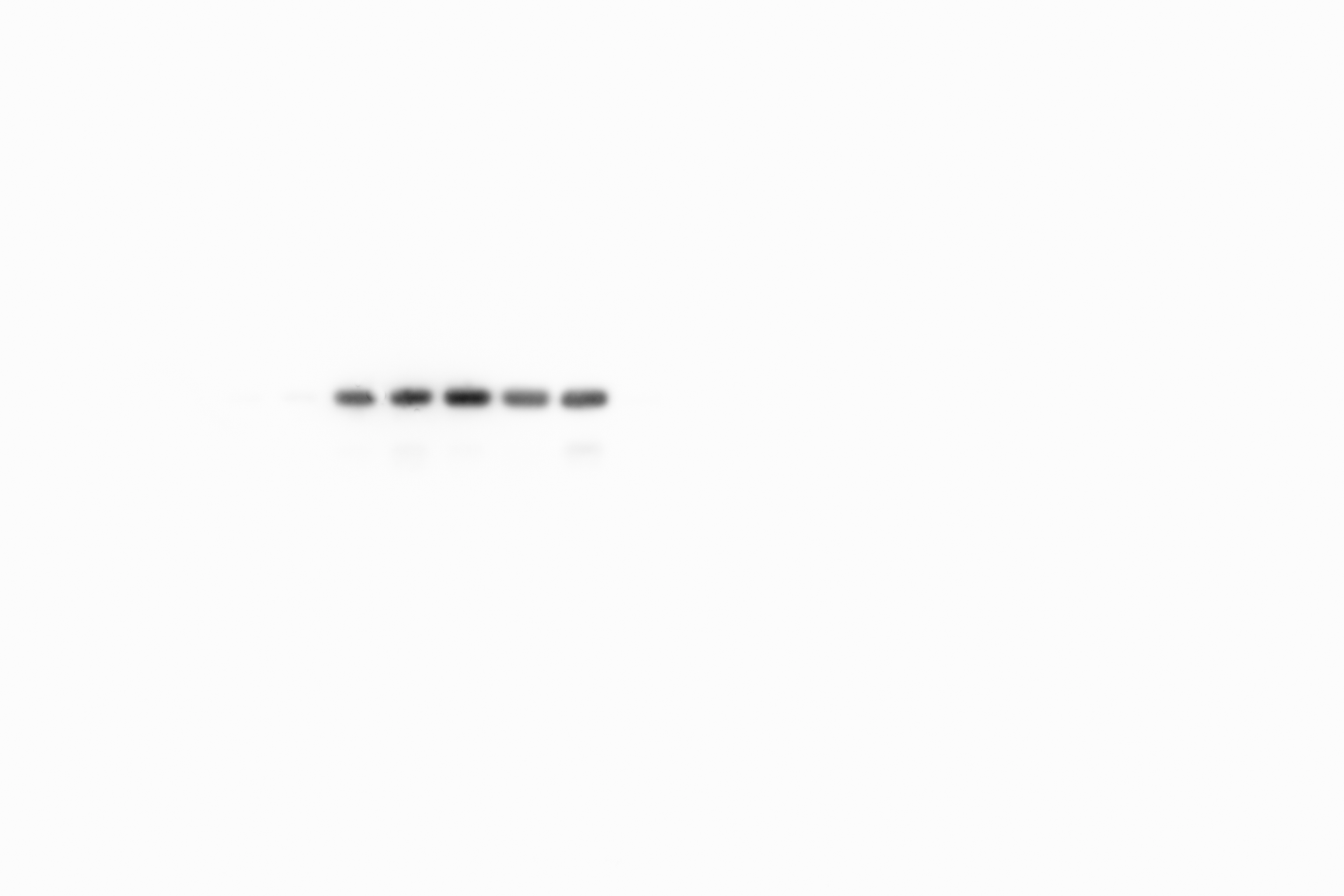

Supplement: Figure 3—source data 1. [file elife-68283-fig3-data1.zip › Figure 3-Source Data 2.tif]

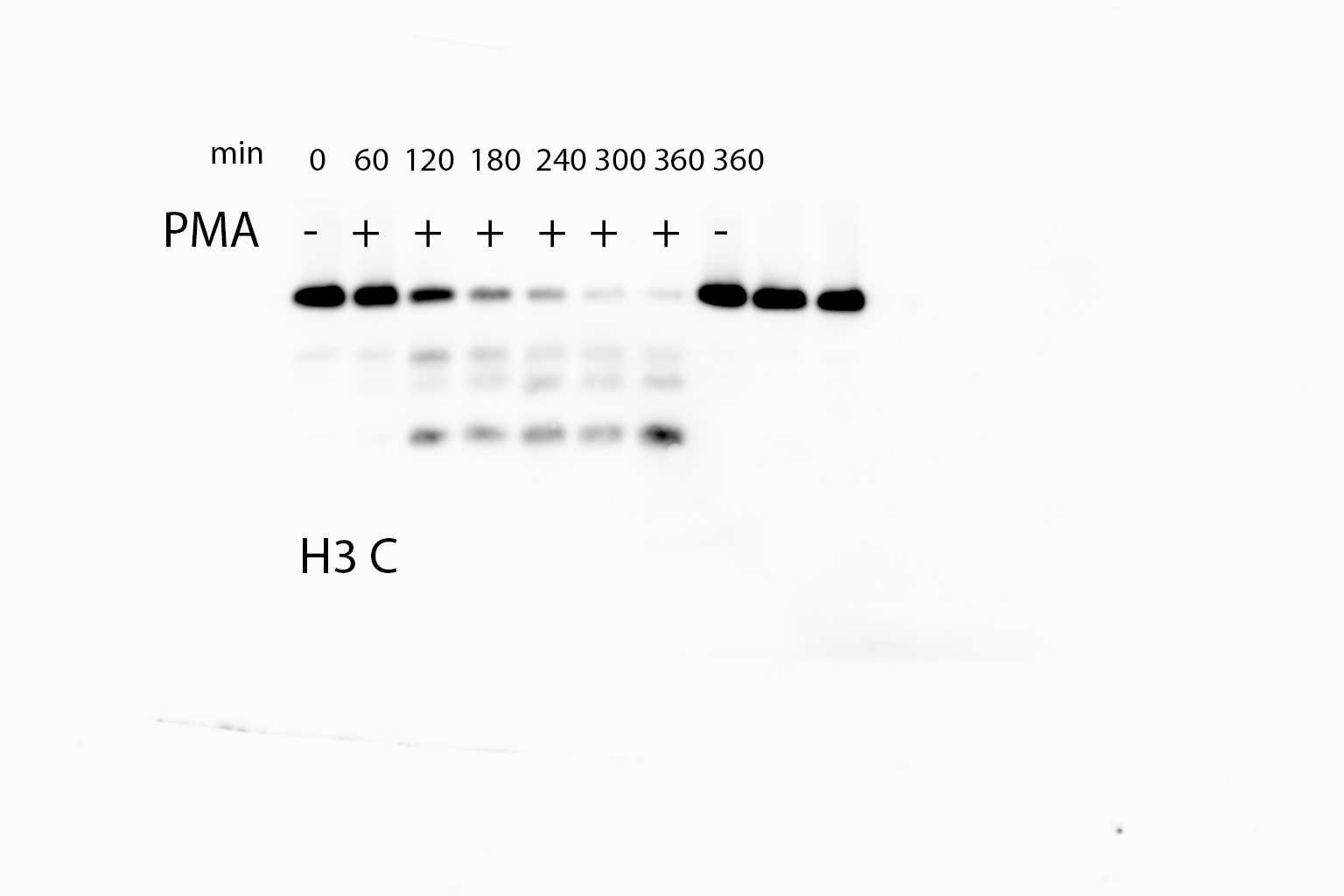

Supplement: Figure 3—source data 1. [file elife-68283-fig3-data1.zip › Figure 3-Source Data 1 copy_annotated 8bit.tif]

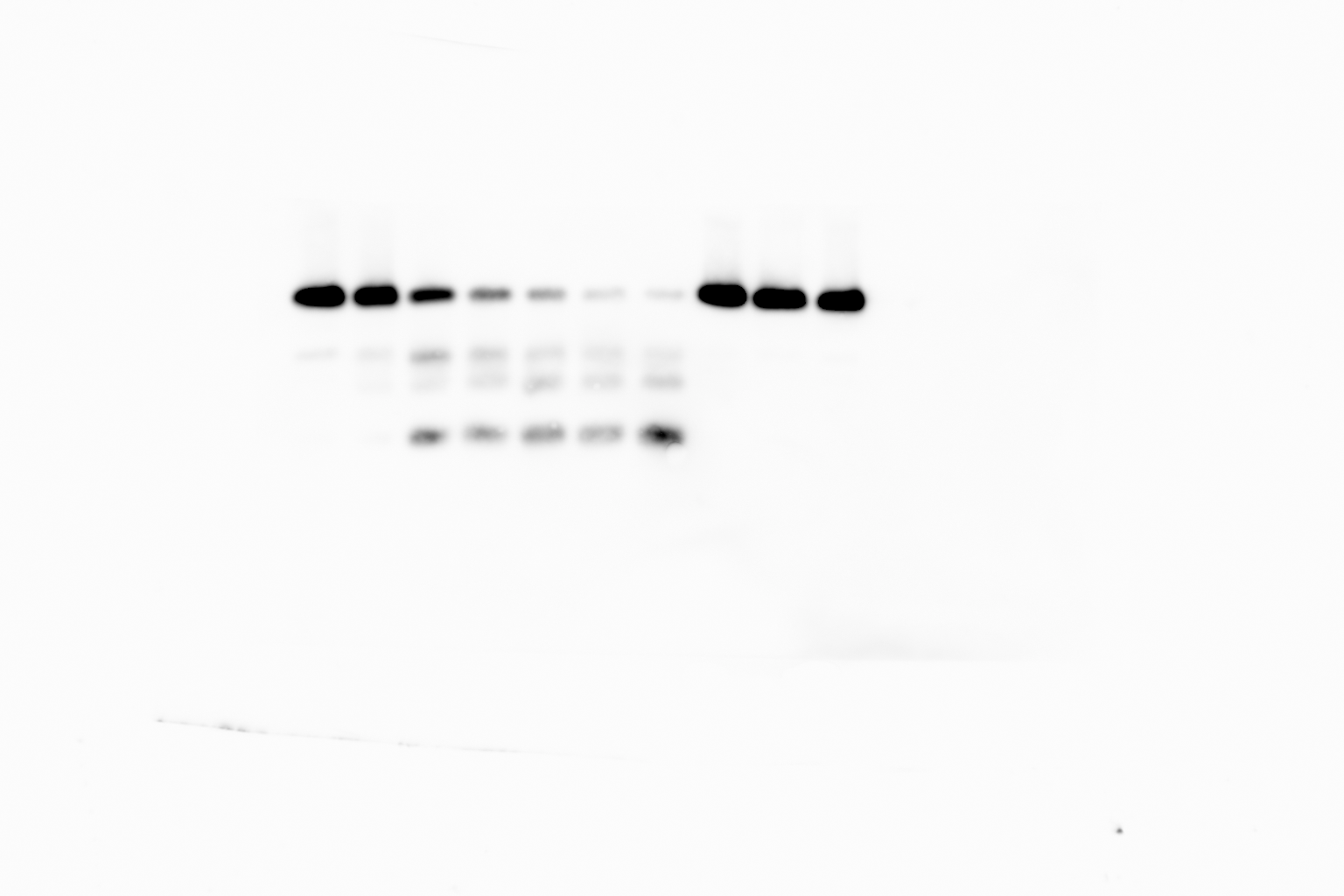

Supplement: Figure 3—source data 1. [file elife-68283-fig3-data1.zip › Figure 3-Source Data 1.tif]

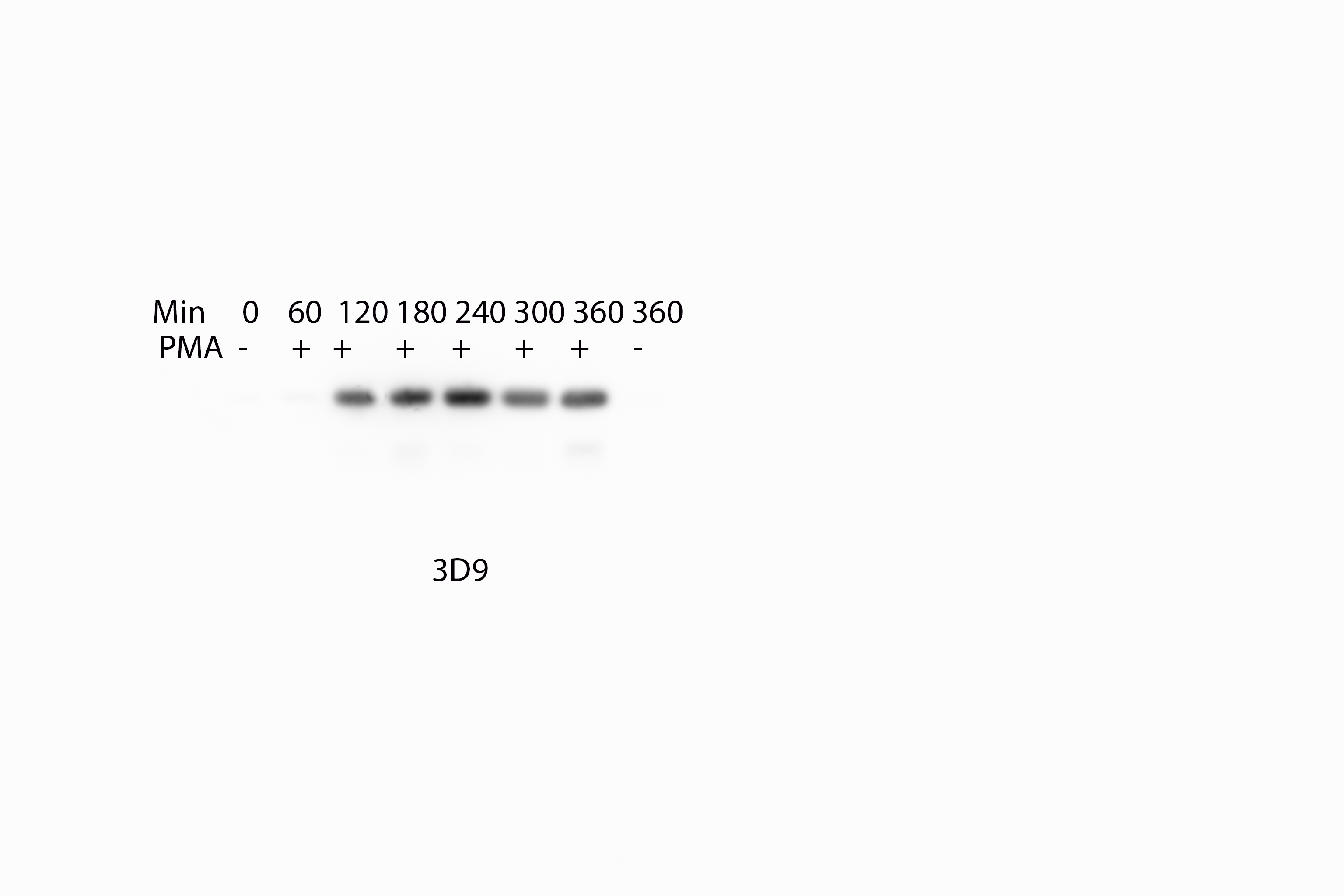

Supplement: Figure 3—source data 1. [file elife-68283-fig3-data1.zip › Figure 3-Source Data 2 copy_annotated.tif]

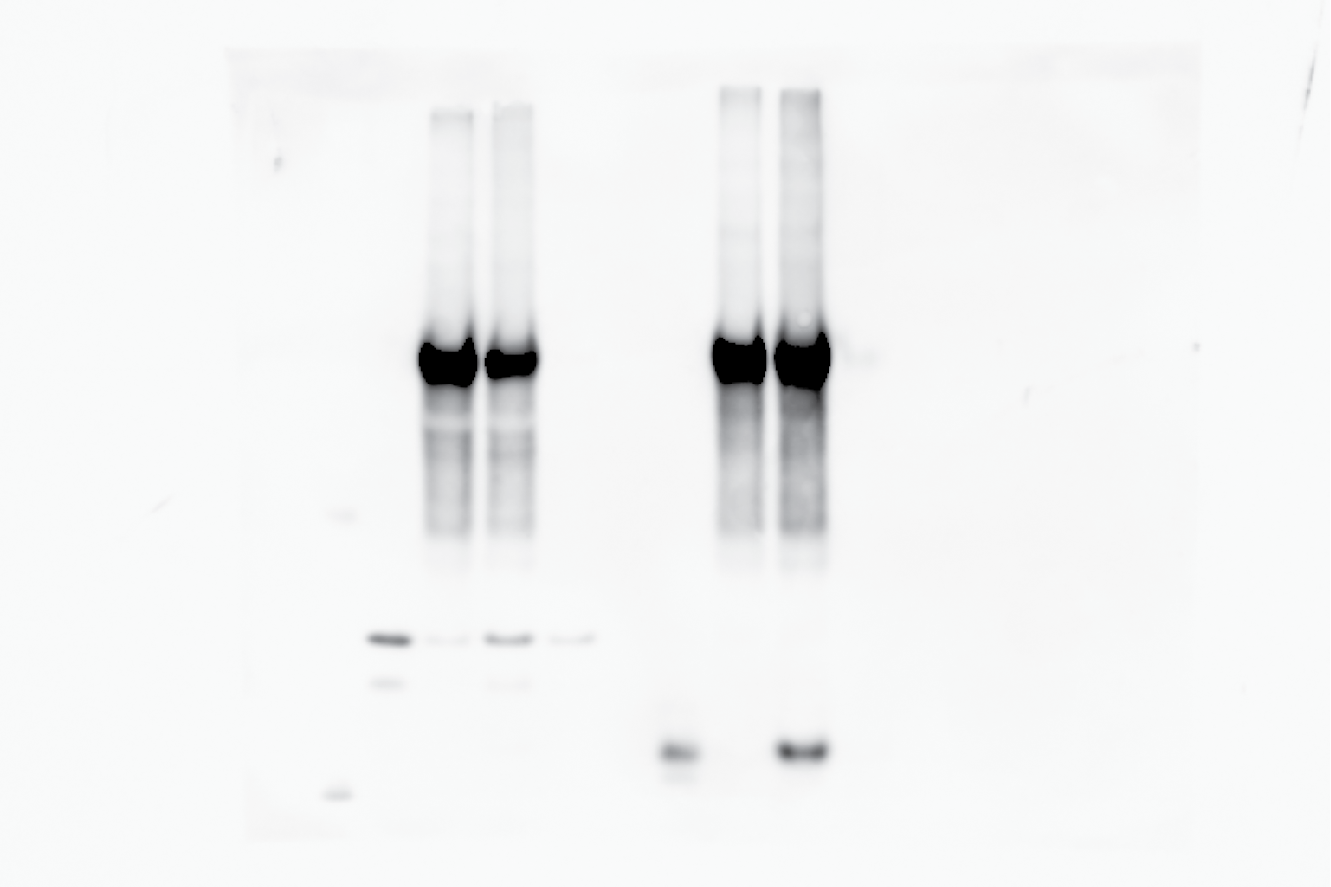

Supplement: Figure 3—figure supplement 3—source data 1. [file elife-68283-fig3-figsupp3-data1.zip › Source Data/2_day2_IP_H3C_high-incr_10s_8_8bit_RGB.tif]

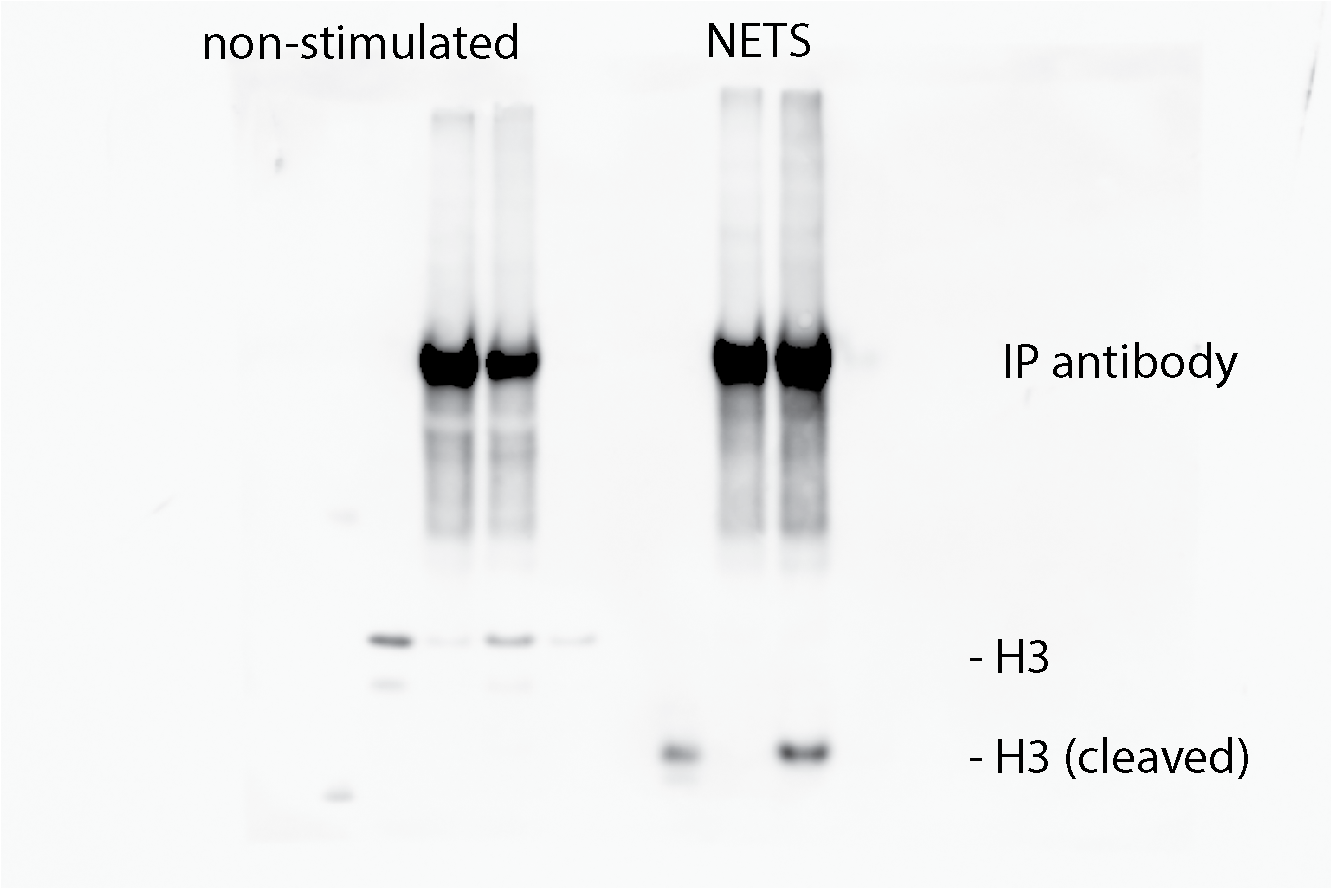

Supplement: Figure 3—figure supplement 3—source data 1. [file elife-68283-fig3-figsupp3-data1.zip › Source Data/2_day2_IP_H3C_high-incr_10s_8_8bit_RGB_annotated.tif]

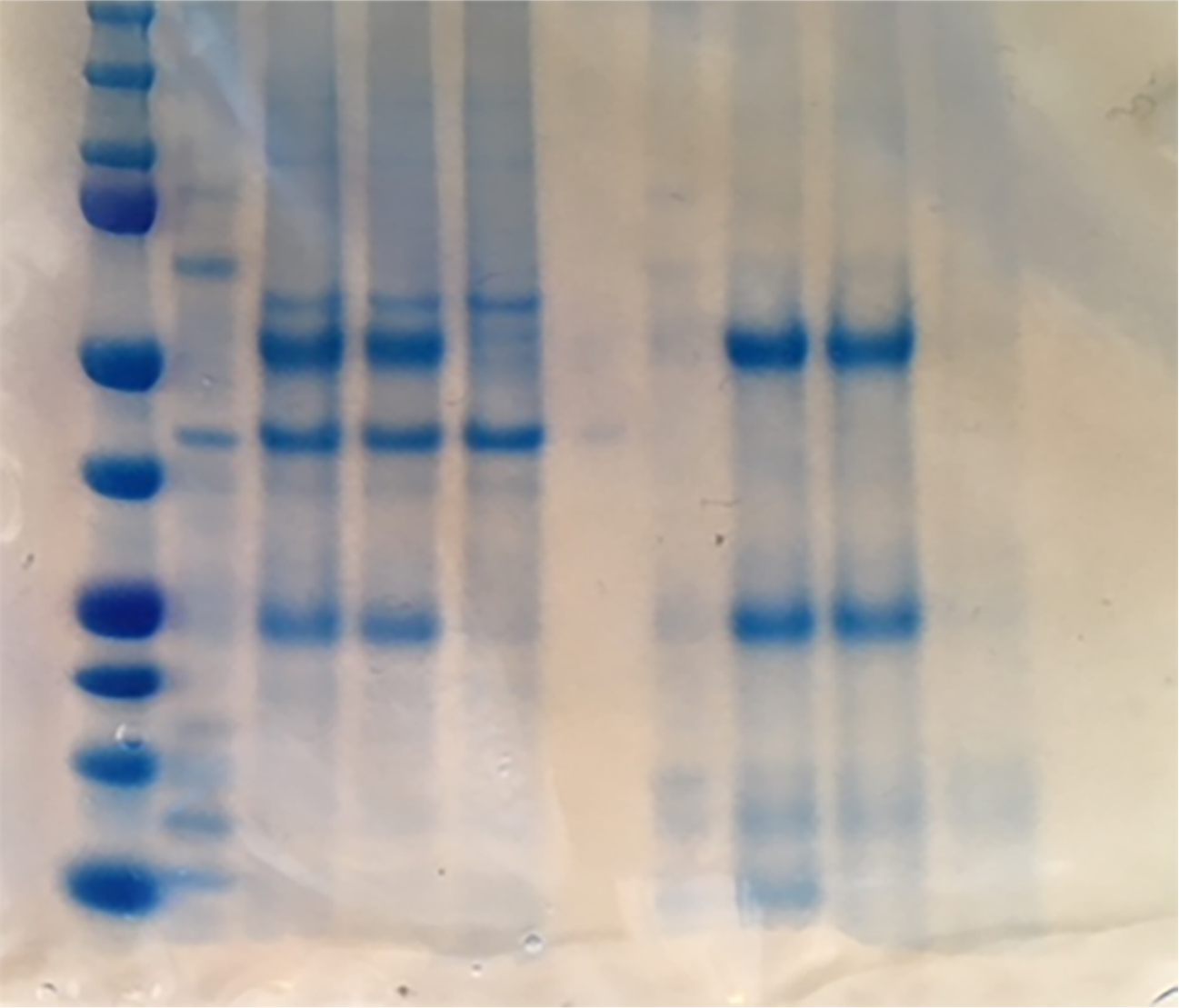

Supplement: Figure 3—figure supplement 3—source data 1. [file elife-68283-fig3-figsupp3-data1.zip › Source Data/i gel_8bit_RGB.tif]

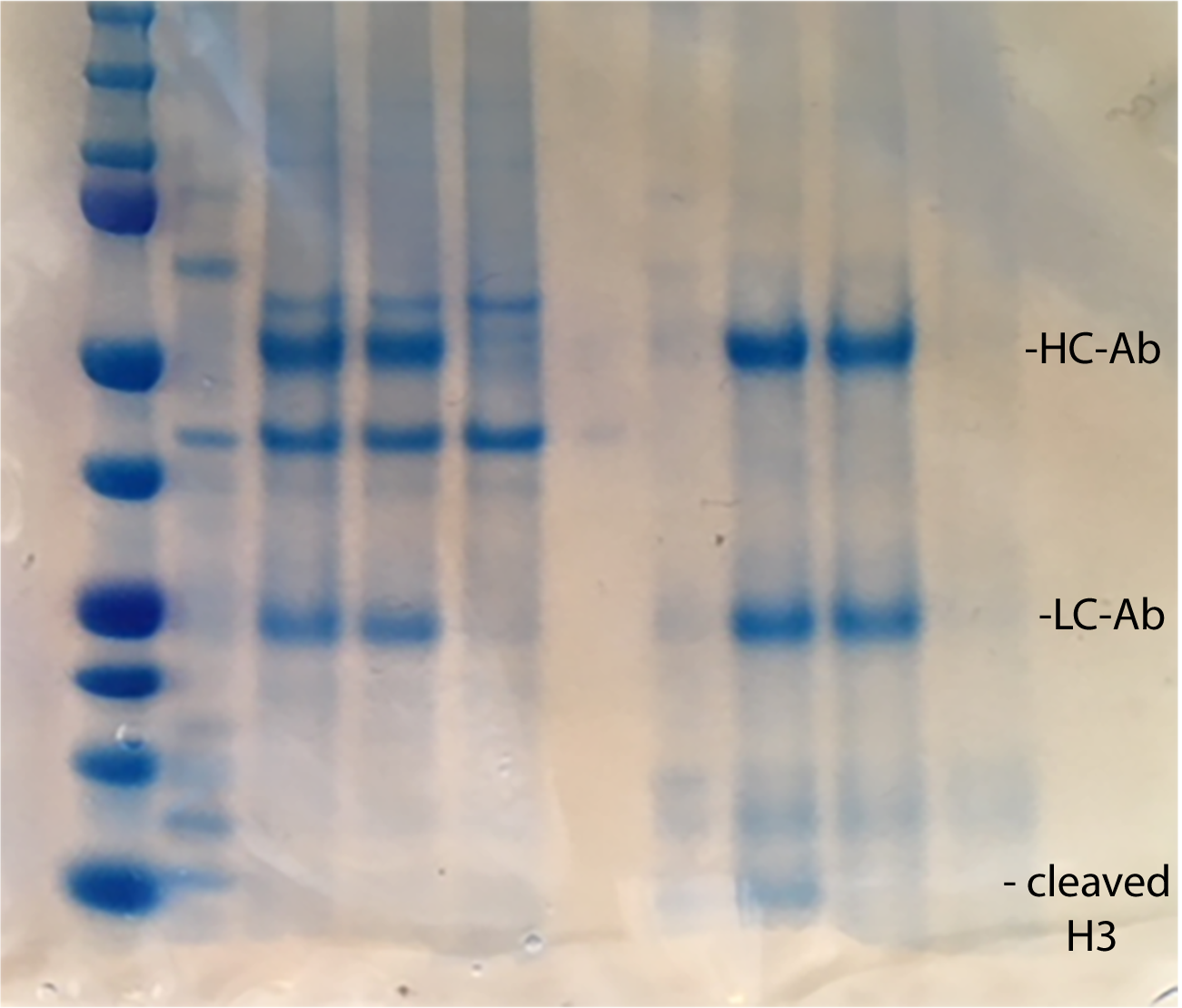

Supplement: Figure 3—figure supplement 3—source data 1. [file elife-68283-fig3-figsupp3-data1.zip › Source Data/i gel_8bit_RGB_annnotated.tif]

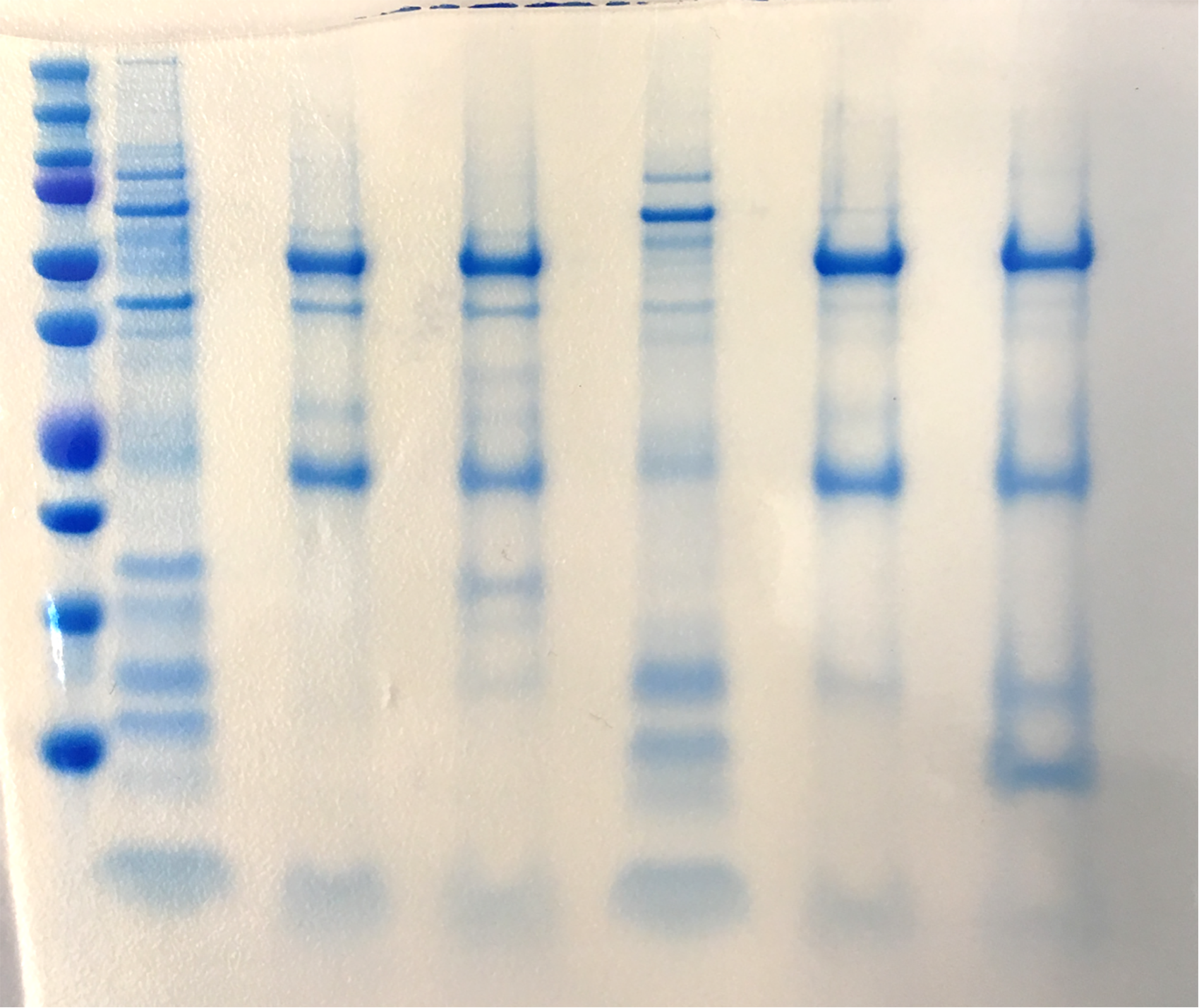

Supplement: Figure 3—figure supplement 3—source data 1. [file elife-68283-fig3-figsupp3-data1.zip › Source Data/ii gel_8bit_RGB.tif]

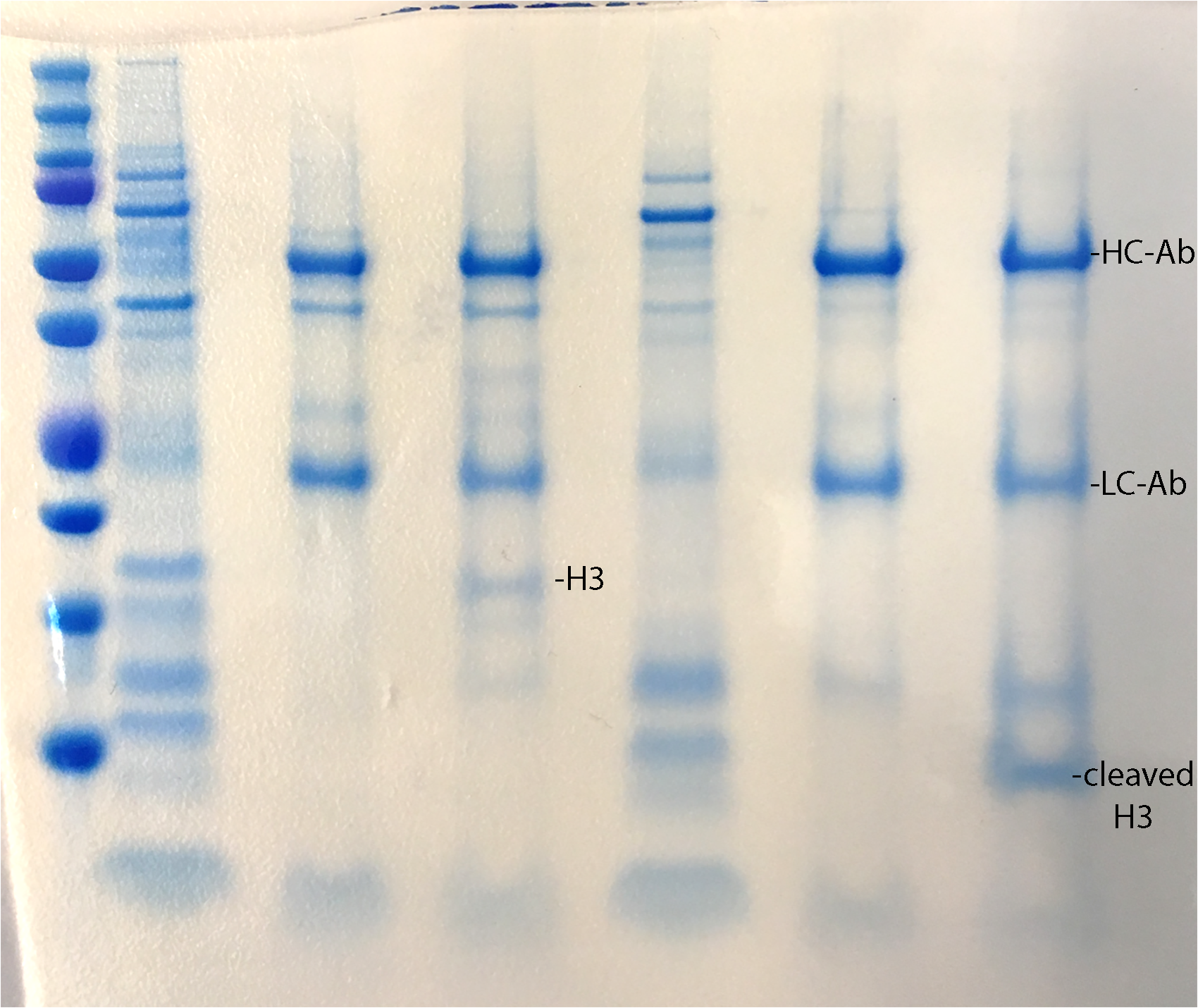

Supplement: Figure 3—figure supplement 3—source data 1. [file elife-68283-fig3-figsupp3-data1.zip › Source Data/ii gel_8bit_RGB_annotated.tif]

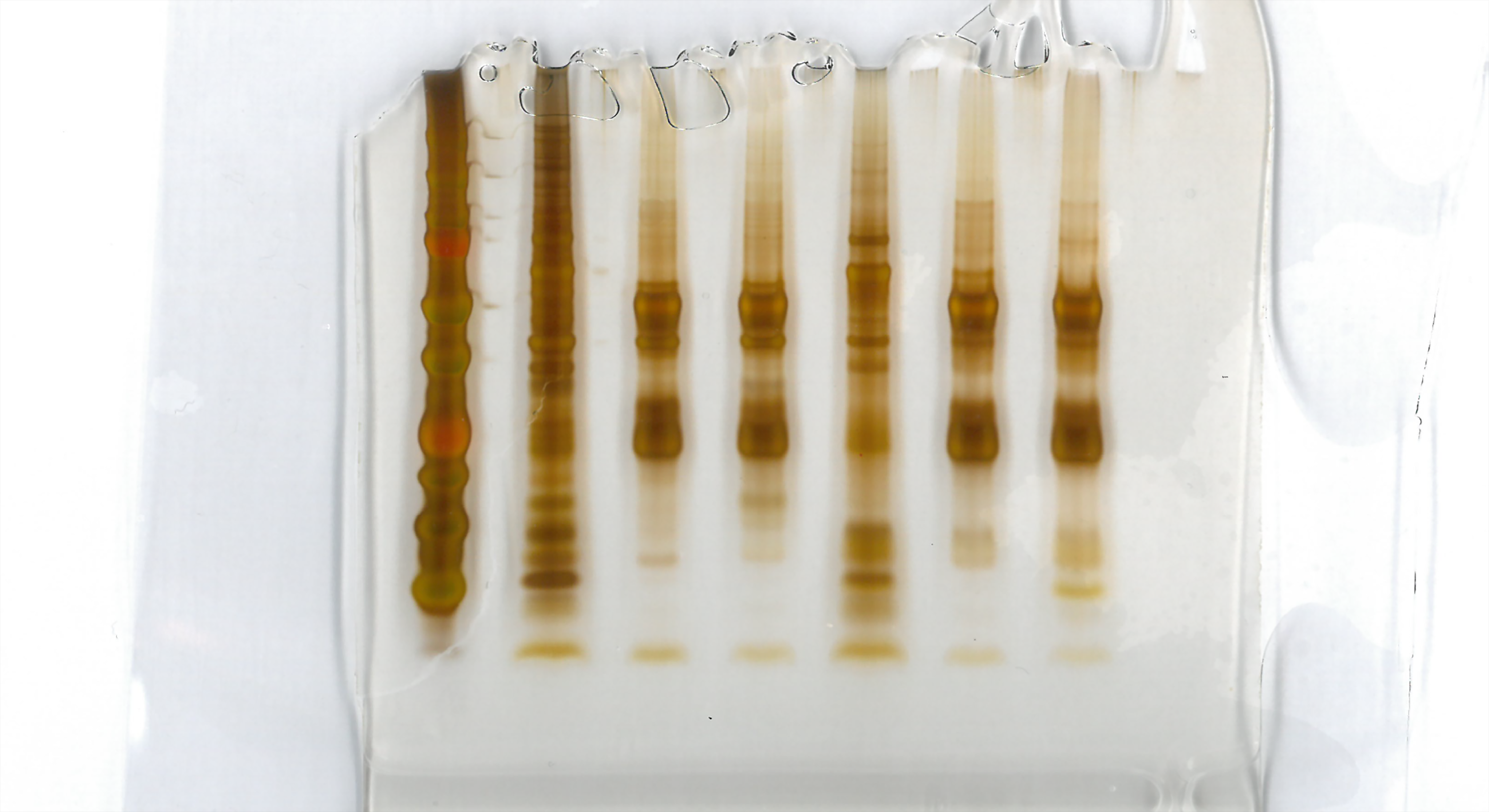

Supplement: Figure 3—figure supplement 3—source data 1. [file elife-68283-fig3-figsupp3-data1.zip › Source Data/silverstain.tif]

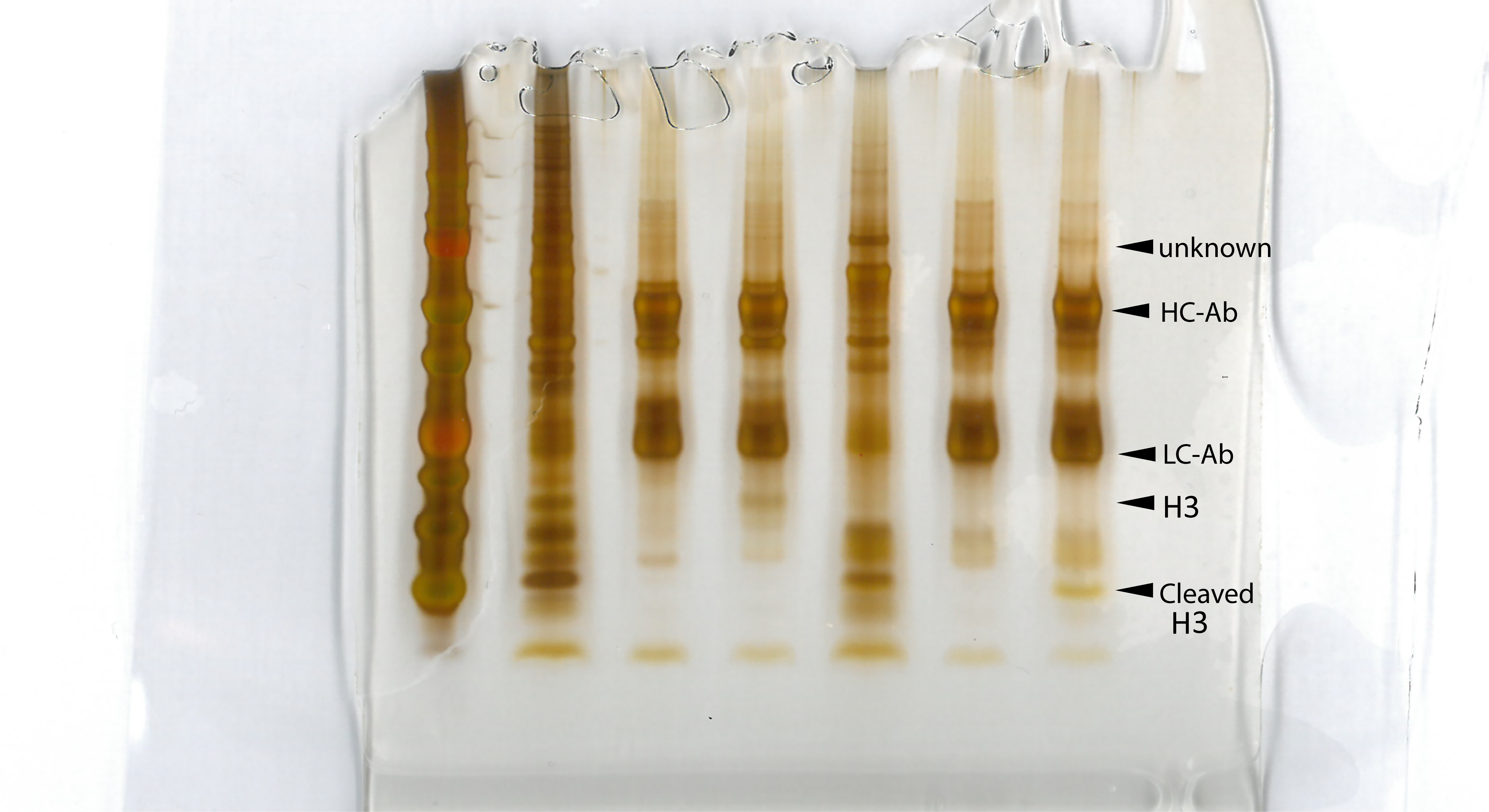

Supplement: Figure 3—figure supplement 3—source data 1. [file elife-68283-fig3-figsupp3-data1.zip › Source Data/silverstain_annotated.tif]

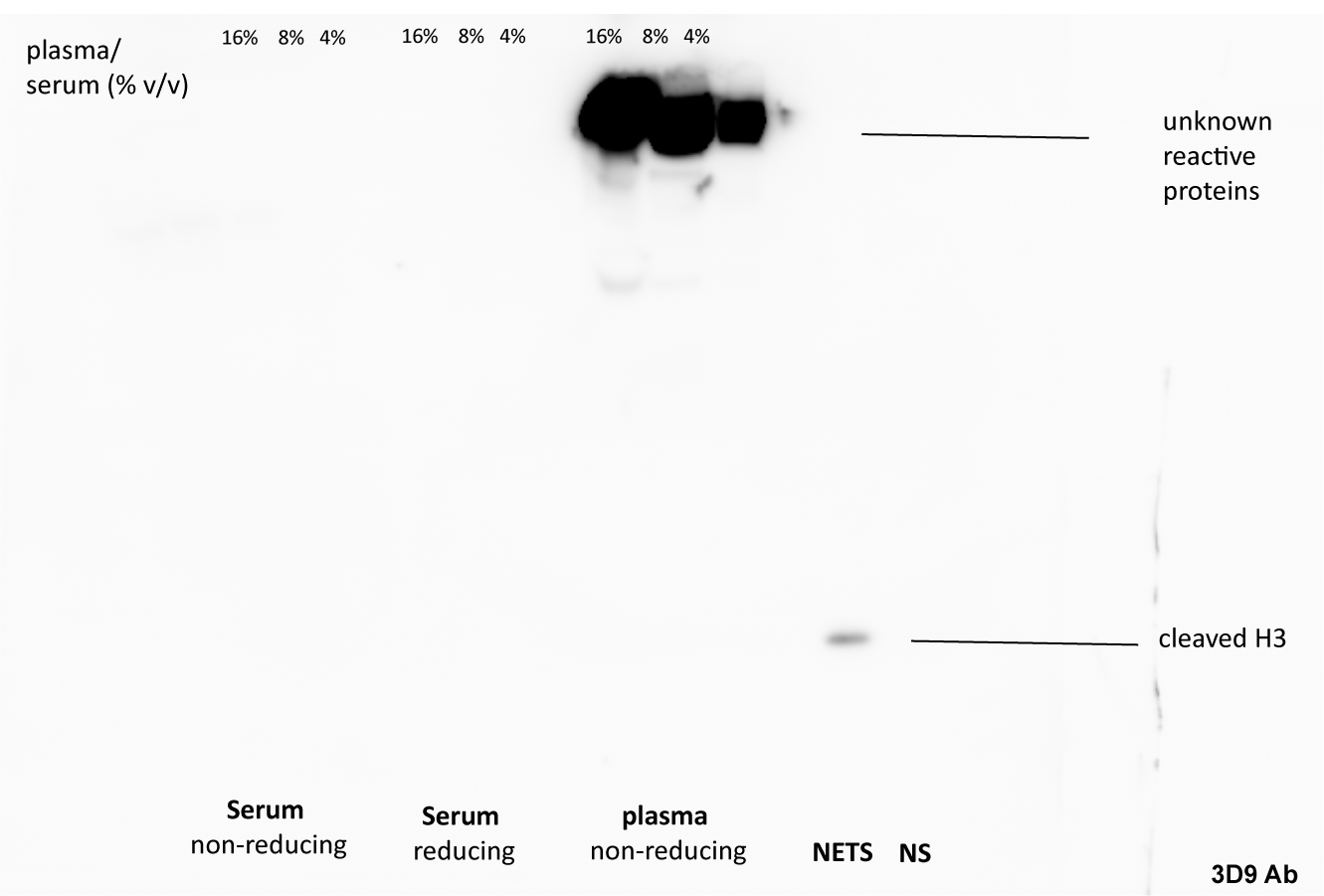

Supplement: Figure 3—figure supplement 4—source data 1. [file elife-68283-fig3-figsupp4-data1.zip › Source data/1_3D9-high_incr-10s_2 annotated.tif]

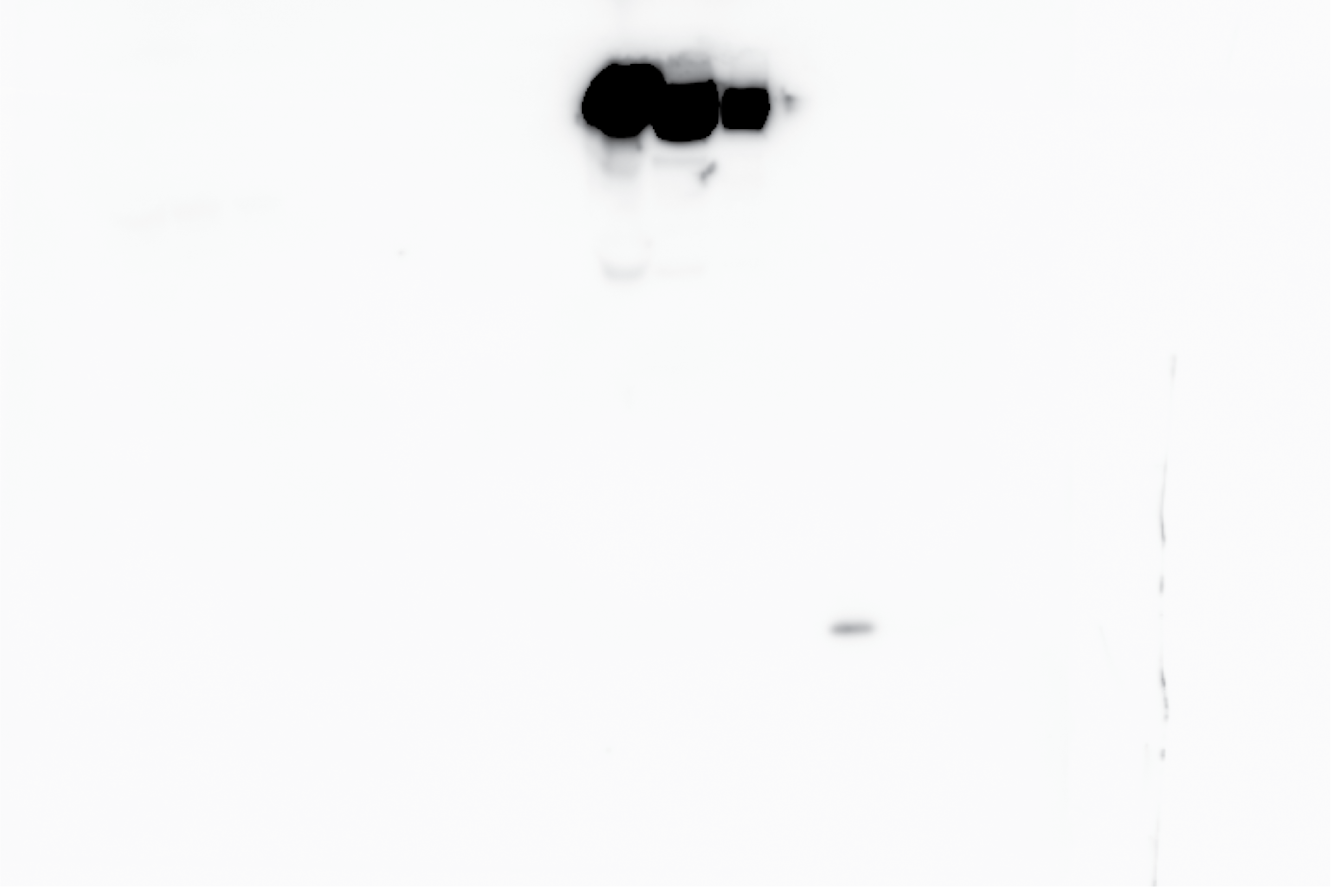

Supplement: Figure 3—figure supplement 4—source data 1. [file elife-68283-fig3-figsupp4-data1.zip › Source data/1_3D9-high_incr-10s_2_8bitRGB.tif]

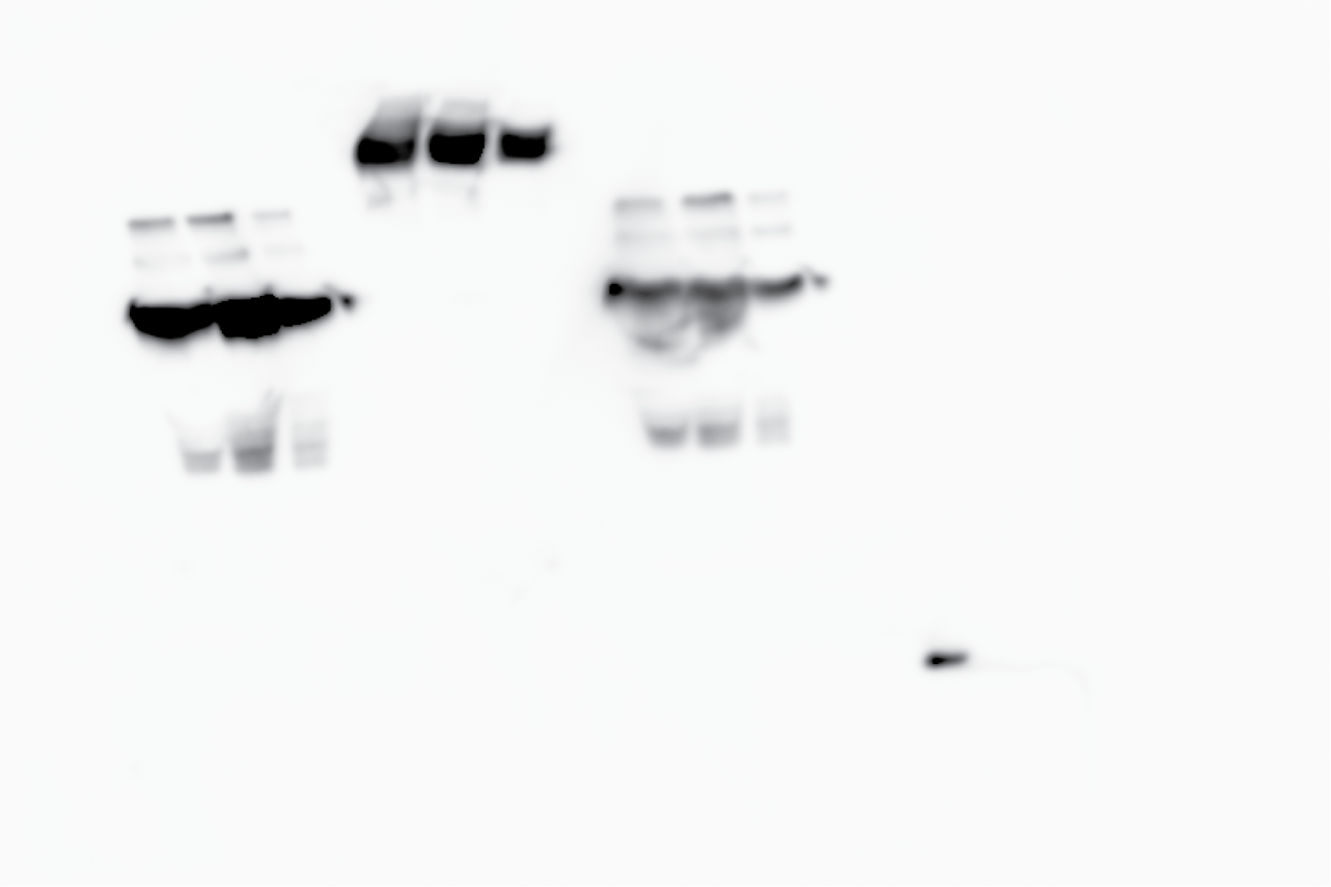

Supplement: Figure 3—figure supplement 4—source data 1. [file elife-68283-fig3-figsupp4-data1.zip › Source data/2_3D9-high_incr-10s_4_8bitRGB.tif]

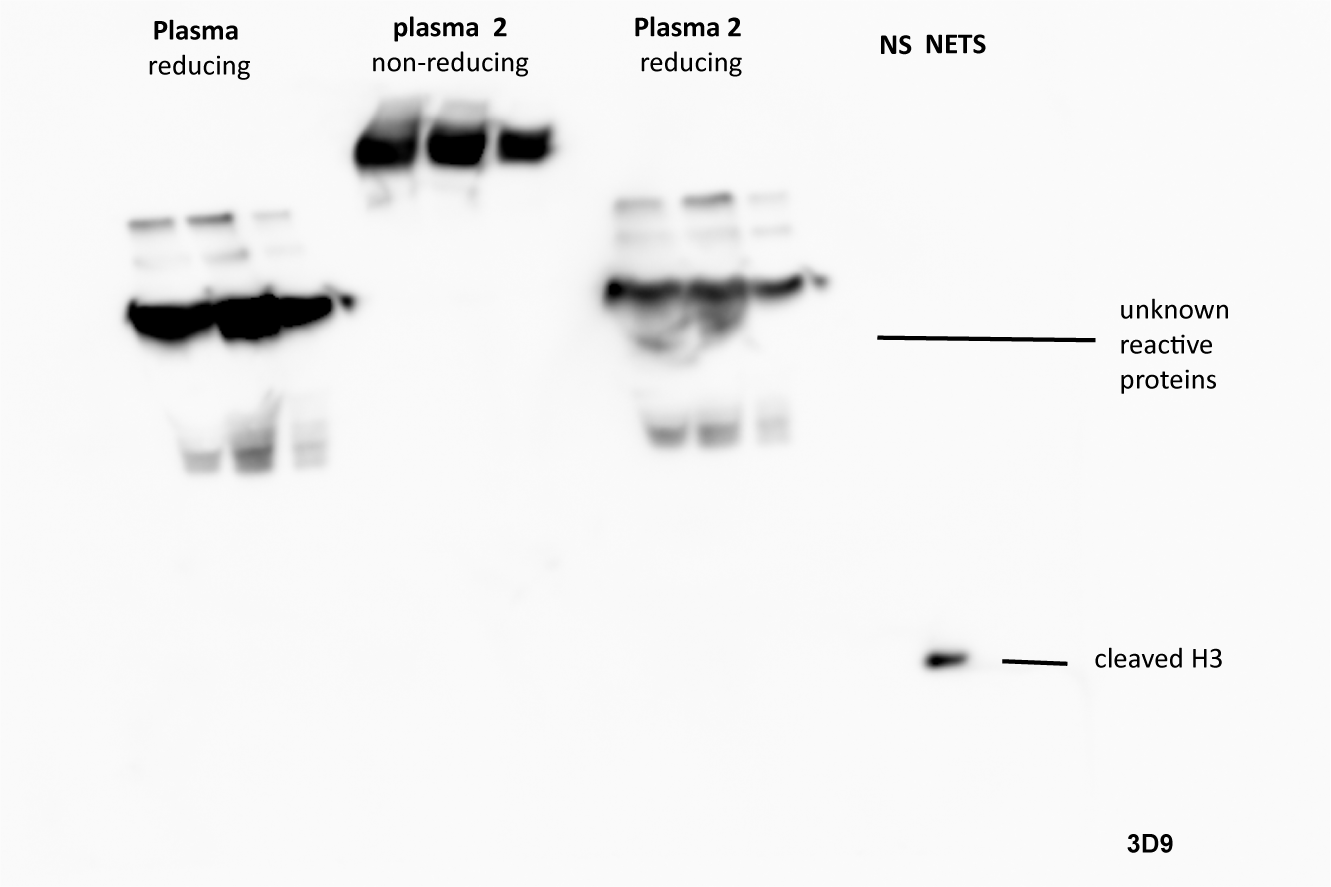

Supplement: Figure 3—figure supplement 4—source data 1. [file elife-68283-fig3-figsupp4-data1.zip › Source data/2_3D9-high_incr-10s_8bitRGB_annotated.tif]

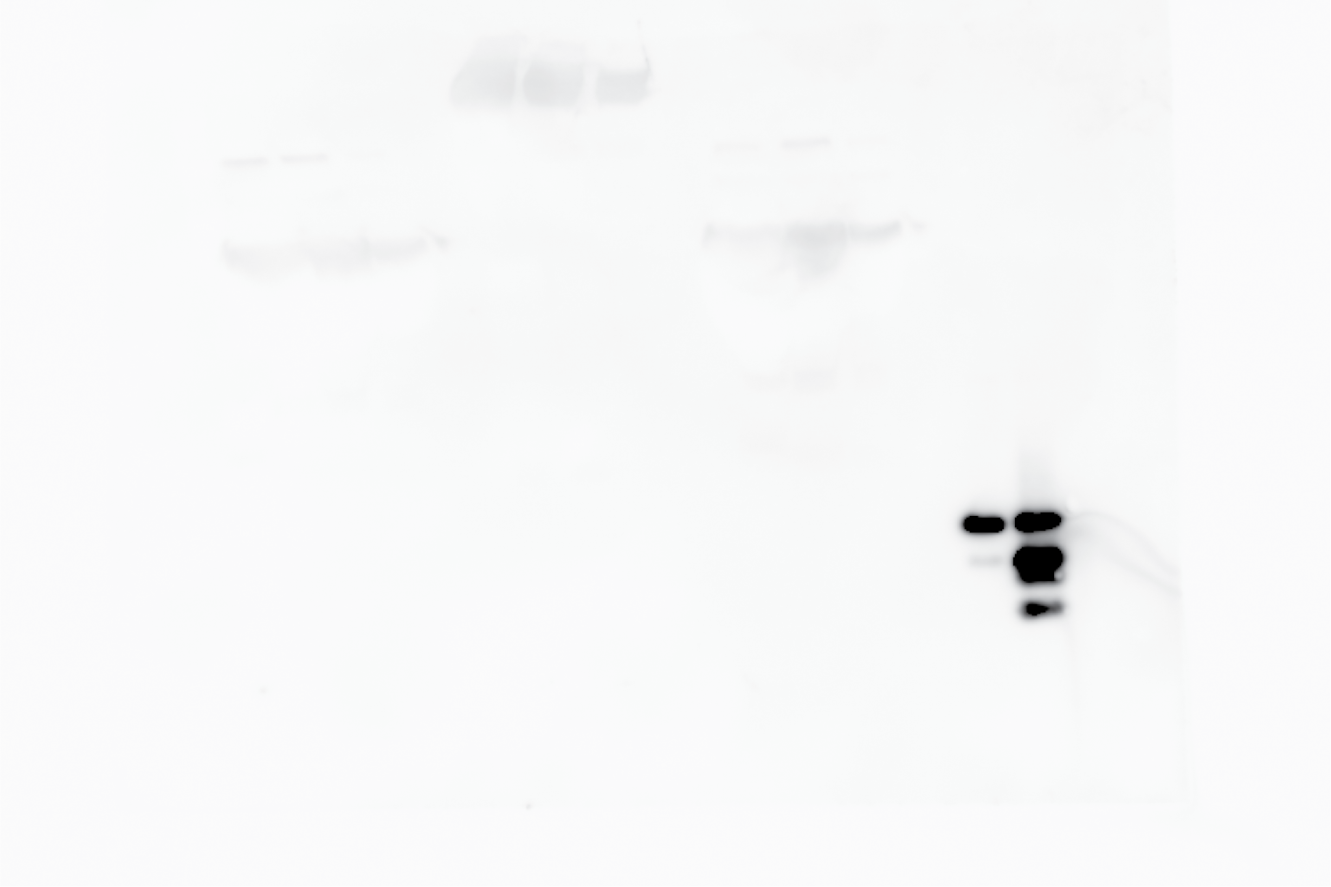

Supplement: Figure 3—figure supplement 4—source data 1. [file elife-68283-fig3-figsupp4-data1.zip › Source data/A_h3C-incr-10s-high_2_8bit_RGB.tif]

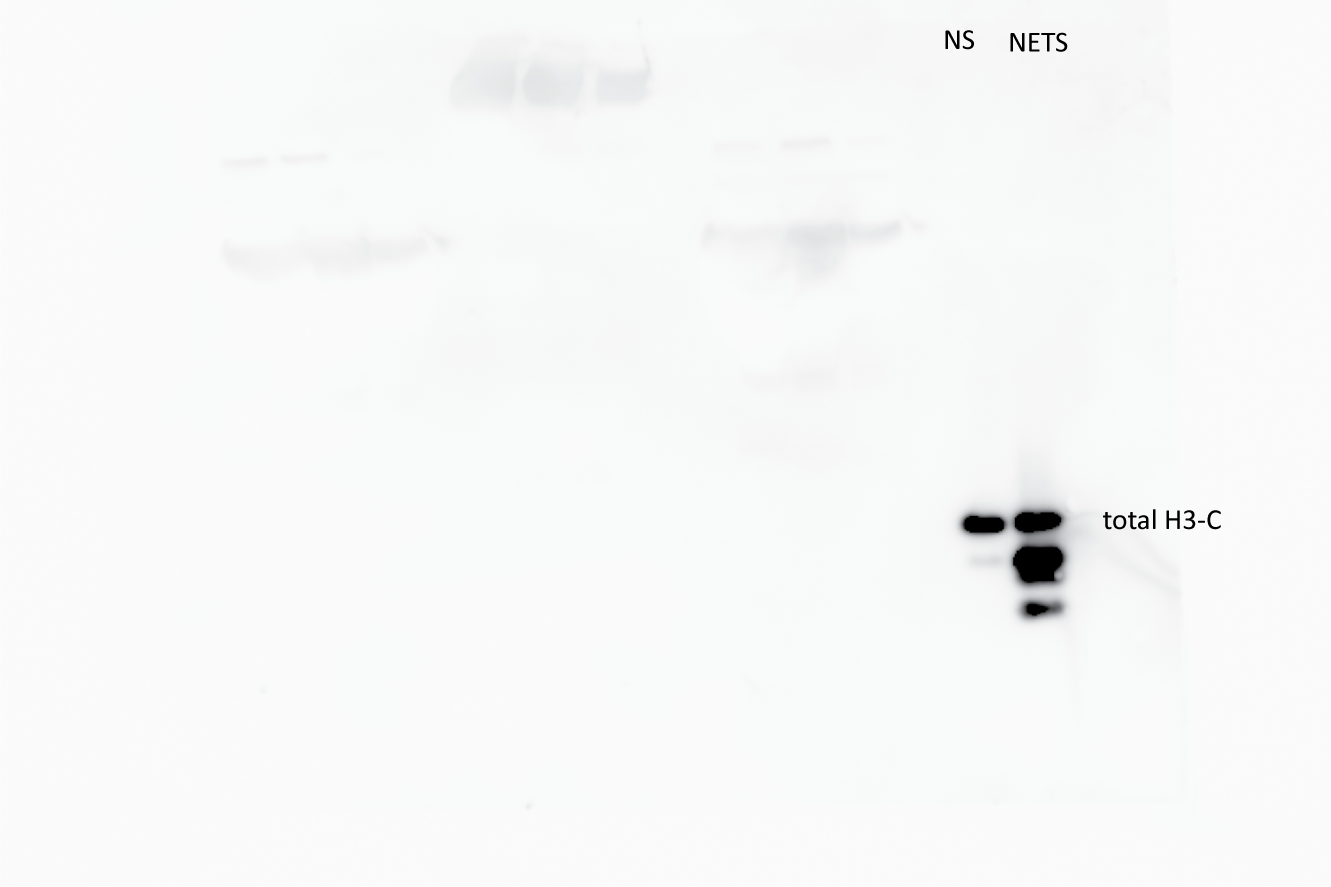

Supplement: Figure 3—figure supplement 4—source data 1. [file elife-68283-fig3-figsupp4-data1.zip › Source data/A_h3C-incr-10s-high_2_8bit_RGB_annotated.tif]

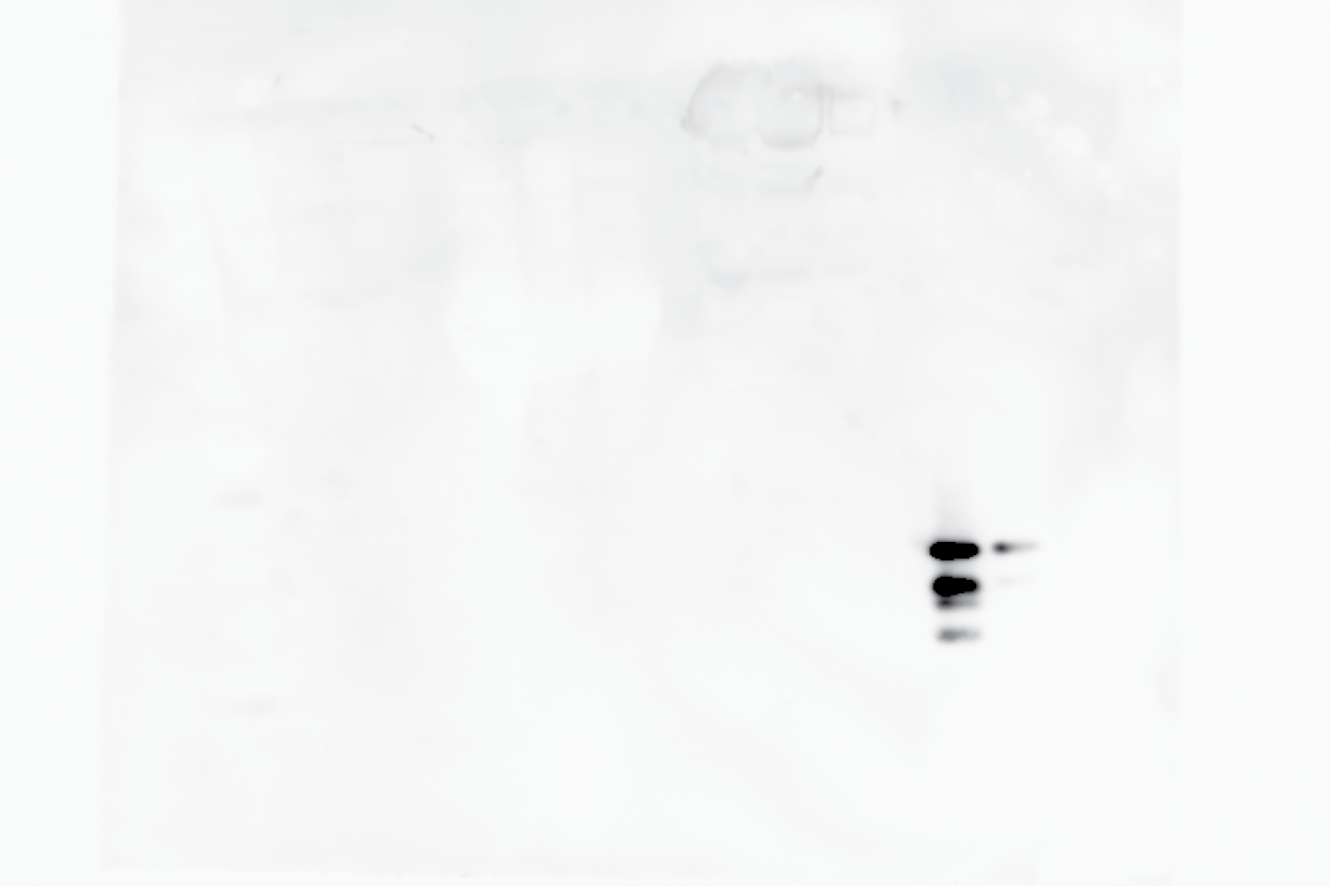

Supplement: Figure 3—figure supplement 4—source data 1. [file elife-68283-fig3-figsupp4-data1.zip › Source data/B_h3C-incr-10s-high_7_8bit_RGB.tif]

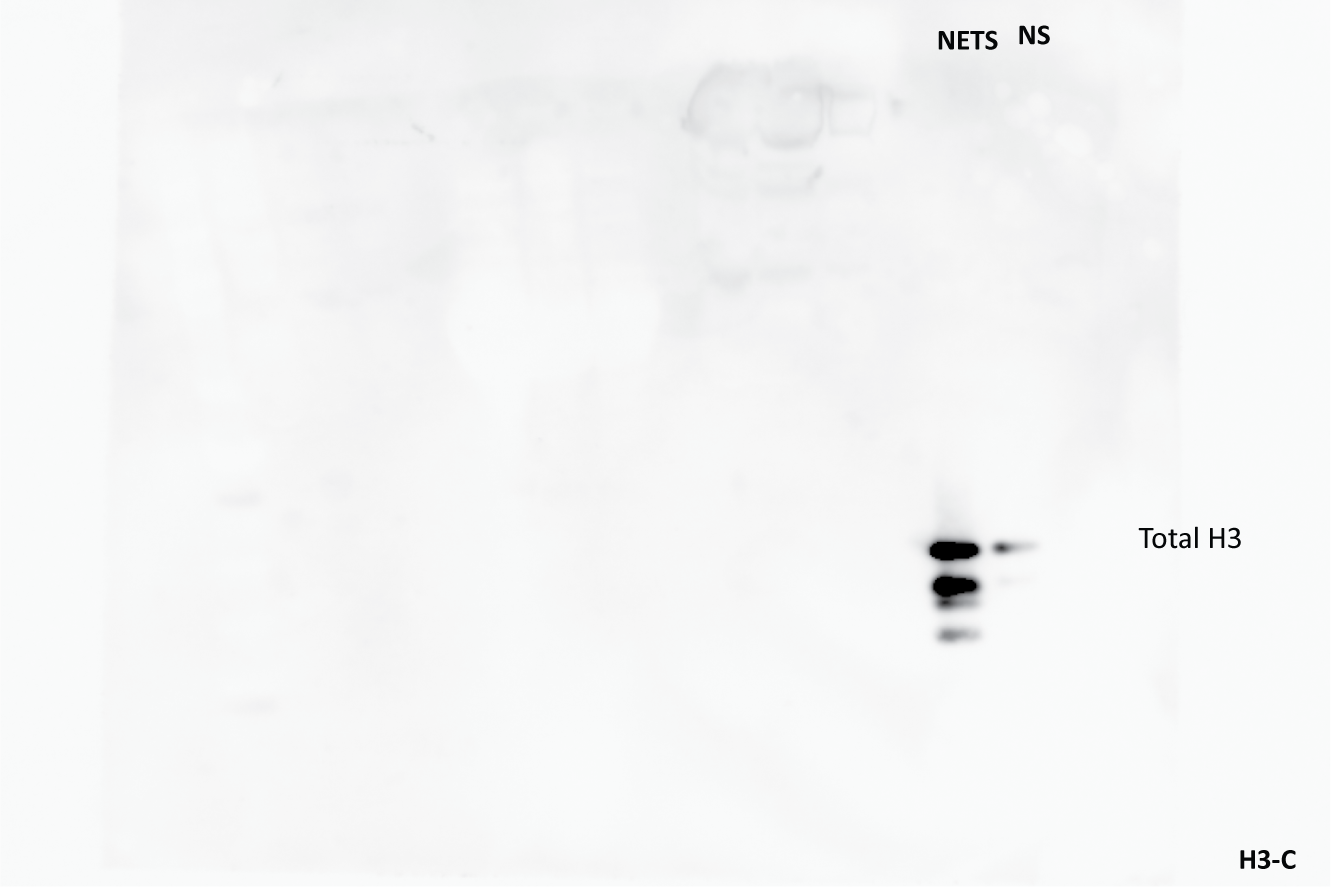

Supplement: Figure 3—figure supplement 4—source data 1. [file elife-68283-fig3-figsupp4-data1.zip › Source data/B_h3C-incr-10s-high_7_8bit_RGB_annotated.tif]
