# Supplementary material for: Histone H3 clipping is a novel signature of human neutrophil extracellular traps
Source: eLife. 2022 Oct 25;11:e68283. doi: 10.7554/eLife.68283 (PMC9665850; doi:10.7554/eLife.68283)
Supplement: Transparent reporting form [file elife-68283-transrepform1.docx]

***eLife’s* transparent reporting form**

We encourage authors to provide detailed information *within their submission* to facilitate the interpretation and replication of experiments. Authors can upload supporting documentation to indicate the use of appropriate reporting guidelines for health-related research (see [EQUATOR Network](http://www.equator-network.org/%20)), life science research (see the [BioSharing Information Resource](https://biosharing.org/)), or the [ARRIVE guidelines](http://www.plosbiology.org/article/info:doi/10.1371/journal.pbio.1000412) for reporting work involving animal research. Where applicable, authors should refer to any relevant reporting standards documents in this form.

If you have any questions, please consult our Journal Policies and/or contact us: [editorial@elifesciences.org](mailto:editorial@elifesciences.org).

**Sample-size estimation**

- You should state whether an appropriate sample size was computed when the study was being designed
- You should state the statistical method of sample size computation and any required assumptions
- If no explicit power analysis was used, you should describe how you decided what sample (replicate) size (number) to use

Please outline where this information can be found within the submission (e.g., sections or figure legends), or explain why this information doesn’t apply to your submission:

All information pertaining to sample size or replicate number can be found in the figure legends. No explicit power analysis was used in the design of this study as it was an explorative study. Experiments were repeated between 3 and 5 times as results were consistent between replications.

**Replicates**

- You should report how often each experiment was performed
- You should include a definition of biological versus technical replication
- The data obtained should be provided and sufficient information should be provided to indicate the number of independent biological and/or technical replicates
- If you encountered any outliers, you should describe how these were handled
- Criteria for exclusion/inclusion of data should be clearly stated
- High-throughput sequence data should be uploaded before submission, with a private link for reviewers provided (these are available from both GEO and ArrayExpress)

Please outline where this information can be found within the submission (e.g., sections or figure legends), or explain why this information doesn’t apply to your submission:

How many times each experiment was repeated is found in Statistics section of the methods and defined for each figure in the figure legends. A statement to define the biological replicate is included in the methods section- Experimental repeats are biological replicates, where each replicate represents cells/NETs isolated from a different donor. In some instances, in ELISA, ROS and cytotoxicity assays, it is explicitly stated in the methods section that technical replicates (duplicates or triplicates) were performed during one biological replicate of an experiment. All results were included where possible. Where obvious errors were made in experimental setup samples were discarded but are still presented in the Source Data but it is indicated that they were left out of the analysis.

**Statistical reporting**

- Statistical analysis methods should be described and justified
- Raw data should be presented in figures whenever informative to do so (typically when N per group is less than 10)
- For each experiment, you should identify the statistical tests used, exact values of N, definitions of center, methods of multiple test correction, and dispersion and precision measures (e.g., mean, median, SD, SEM, confidence intervals; and, for the major substantive results, a measure of effect size (e.g., Pearson's r, Cohen's d)
- Report exact p-values wherever possible alongside the summary statistics and 95% confidence intervals. These should be reported for all key questions and not only when the p-value is less than 0.05.

Please outline where this information can be found within the submission (e.g., sections or figure legends), or explain why this information doesn’t apply to your submission:

This study attempted to describe a biological event – histone clipping in NET formation - and then characterise the behaviour of a new antibody, thus only descriptive statistics (N, mean or median plus/minus standard deviation were used where relevant and are outlined in the figure legend. No statistical tests were applied.

(For large datasets, or papers with a very large number of statistical tests, you may upload a single table file with tests, Ns, etc., with reference to sections in the manuscript.)

**Group allocation**

- Indicate how samples were allocated into experimental groups (in the case of clinical studies, please specify allocation to treatment method); if randomization was used, please also state if restricted randomization was applied
- Indicate if masking was used during group allocation, data collection and/or data analysis

Please outline where this information can be found within the submission (e.g., sections or figure legends), or explain why this information doesn’t apply to your submission:

No masking or randomisation was used for group allocation. All donors were considered to be healthy and not further divided by age or sex.

**Additional data files (“source data”)**

- We encourage you to upload relevant additional data files, such as numerical data that are represented as a graph in a figure, or as a summary table
- Where provided, these should be in the most useful format, and they can be uploaded as “Source data” files linked to a main figure or table
- Include model definition files including the full list of parameters used
- Include code used for data analysis (e.g., R, MatLab)
- Avoid stating that data files are “available upon request”

Please indicate the figures or tables for which source data files have been provided:

Source data files for figures containing graphs have been uploaded. Source data files containing original blots and gels, and annotated originals are provided.

Figure 1 – Source Data 1

Figure 1 – Figure supplement 1 – Source Data 1

Figure 1 – Figure supplement 2 – Source Data 1

Figure 1 – Figure supplement 3 – Source Data 1

Figure 2 – Source Data 1

Figure 2 – Source Data 2

Figure 2 – Source Data 3

Figure 2 – figure supplement 2 - Source Data 1

Figure 3 - Source Data 1

Figure 3 - Source Data 2

Figure 3-Figure supplement 3 – Source Data 1

Figure 3-Figure supplement 4 – Source Data 1

Figure 3-Figure supplement 4 – Source Data 2

Figure 4-Figure supplement 1 - Source Data 1

Figure 4-Figure supplement 2 - Source Data 1

Figure 4-Figure supplement 3 - Source Data 1

Figure 5 - Source Data 1

Figure 5 - Source Data 2

Figure 5 - Source Data 3

Code for analysis of microscopy has been deposited at the link provided in the text. Subsequent to publication the hybridoma will be deposited in a public access archive.
